# Stable and Efficient Agrobacterium-Mediated Genetic Transformation of Larch Using Embryogenic Callus

**DOI:** 10.3389/fpls.2020.584492

**Published:** 2020-11-25

**Authors:** Yue Song, Xiaoming Bai, Shiwei Dong, Yuning Yang, Hao Dong, Nairui Wang, Hanguo Zhang, Shujuan Li

**Affiliations:** State Key laboratory of Tree Genetics and Breeding, Northeast Forestry University, Harbin, China

**Keywords:** conifers, *Larix*, *Larix olgensis*, somatic embryogenesis, synchronized culture, transgenic, GUS

## Abstract

*Larix olgensis* or larch is an economically important coniferous tree species with rapid growth in the early stages, strong adaptability, and a short time to harvest. The genetic improvement of larch has garnered considerable attention in recent years for reclaiming timber forests. However, traditional breeding methods are largely ineffective for achieving rapid genetic improvement of *L. olgensis*. Studies show that the efficiency of plant regeneration can be improved by optimizing somatic embryogenesis. On this basis, we devised a stable, fast and efficient *Agrobacterium*-mediated genetic transformation method using suspended embryogenic calluses as explants and β-glucuronidase as the reporter. We evaluated the effects of the *Agrobacterium* load, co-culture period, and addition of acetosyringone and transformant screening antibiotic on the transformation efficiency. In addition, we tested the pCAMBIA 1300-*Pt*HCA 2-1 promoter-GUS binary expression vector, which contains the GUS gene ORF under the control of *Populus trichocarpa* high cambial activity *Pt*HCA 2-1 promoter, and observed the tissue-specific expression of the GUS gene in the somatic embryos of transgenic larch. This novel technique can not only accelerate the generation of superior transgenic strains of *L. olgensis* but also aid in future gene functional studies.

## Introduction

*Larix olgensis* is a coniferous tree distributed across northeastern China, Russia, and Korea, and its timber is used in construction and other industries (Hu et al., 2015). It is an important species for reclaiming timber forests due to its rapid growth at the early stages, strong adaptability, and a short harvesting cycle (Keith and Chauret, 2011; Boruszewski et al., 2017). With a globally dwindling forest cover, there has been an increased focus in recent years to develop genetically improved strains of *Larix* in order to accelerate afforestation. However, as most woody plants, *Larix* has a long juvenile period, and the offspring produced by sexual reproduction show considerable genetic variation. Therefore, traditional breeding methods cannot achieve rapid genetic improvement of *L. olgensis*.

On the other hand, transgenesis is a promising approach for accelerating the genetic improvement of forest trees (Vain et al., 2004; Flachowsky et al., 2009; Ren et al., 2017). The regenerative ability of the recipient parts, such as zygotic embryos, hypocotyl, embryogenic callus, and somatic embryos (Lin et al., 2005; Prakash and Gurumurthi, 2009; Belide et al., 2017), is a major determinant of genetic transformation. The embryogenic callus is an ideal material for transgenesis on account of its stable proliferation, high regeneration rate, sensitivity to screening antibiotics, such as kanamycin and hygromycin, and tolerance to *Agrobacterium tumefaciens* (Belide et al., 2017; Ratjens et al., 2018). Several groups have successfully used embryonic tissues or cellular suspension for genetic transformation of conifers through *A. tumefaciens* (Levee et al., 1997, 1999; Ismail et al., 2004) or gene gun bombardment (Klimaszewska et al., 1997).

*Agrobacterium*-mediated transformation has the advantages of technical ease and stable expression of exogenous genes (Takata and Eriksson, 2012). *Larix* are the natural hosts of *Agrobacterium*, and Huang et al. (1991) first reported the effective genetic transformation of *Larix decidua* seedlings using *Agrobacterium rhizogenes* back in 1991. Subsequently, the transgenic lines of *Larix kaempferi* × *decidua* (Levee et al., 1997), *L. decidua* (Ismail et al., 2004), *L. kaempferi × Larix principis* (Wang, 2007), and *Larix leptolepis* (Zhu et al., 2011) were also established by the *A. tumefaciens*-mediated transformation method (Supplementary Table S1). In addition, gene gun bombardment (Duchesne et al., 1993; Klimaszewska et al., 1997; Qi et al., 2000), pollen tube (Han et al., 2006), and electroporation (Charest et al., 2011) have also been utilized for *Larix* transgenesis, although the transformation efficiency was unsatisfactory.

According to previous reports (Supplementary Table S1), the efficacy of *Agrobacterium*-mediated T-DNA transformation of larch depends on the donor and recipient strains, infection time, co-cultivation time, selection pressure, etc. Moreover, the suitable transformation conditions of different larch species (including interspecific hybrids) are distinct. Our preliminary results showed that the expression rate of exogenous gene in embryogenic callus of *L. olgensis* infected by *A. tumefaciens* using previously established protocols was either very low (less than 5%) or undetectable. Therefore, it is essential to develop a stable and efficient genetic transformation system for *L. olgensis.*

In this study, we have described a novel *Agrobacterium*-based transformation method for *L. olgensis* using embryogenic calluses as the explants. Furthermore, the somatic embryos of transgenic larch transformed with GUS gene ORF under the control of *Populus trichocarpa HCA 2-1* promoter expressed the gene in a tissue-specific manner. This novel transformation system can greatly accelerate the development of genetically improved *L. olgensis* strains.

## Materials and Methods

### Callus Induction

The immature seeds of *L. olgensis* were collected in May 2014 from the Heilongjiang Province. The average size of the embryo proper obtained after peeling the endosperm was 318.04 ± 83.23 μm. The seeds were sterilized using 75% ethanol for 30 s and then 3% sodium hypochlorite for 10 min, cut longitudinally, and then inoculated onto the basic medium (BM; Supplementary Table S2) supplemented with 1.5 mg⋅L^–1^ 2,4-dichlorophenoxyacetic acid (2,4-D), 0.5 mg⋅L^–1^ 6-benzylaminopurine (6-BA), 0.5 mg⋅L^–1^ kinetin (KT), 1.0 g⋅L^–1^ glutamine (Gln), 0.5 g⋅L^–1^ acid hydrolysis casein (CH), 25.0 g⋅L^–1^ sucrose, and 6.0 g⋅L^–1^ agar at pH 6 ± 0.02 (Wang et al., 2009; Song et al., 2016). Callus formation was induced at 23 ± 2°C in the dark. After 7 weeks, the embryogenic calluses were inoculated into liquid proliferation medium (PM) consisting of the basic elements of BM (2,4-D 0.15 mg⋅L^–1^, 6-BA 0.05 mg⋅L^–1^, KT 0.05 mg⋅L^–1^, Gln 1.0 g⋅L^–1^, CH 0.5 g⋅L^–1^ and sucrose 25.0 g⋅L^–1^) at the calluses/medium ratio of 1:100 (w/v). The cultures were maintained with constant shaking at 120 rpm and passaged every 14 days.

### Callus Synchronization and Maturation of Somatic Embryos

**FIGURE 1:**
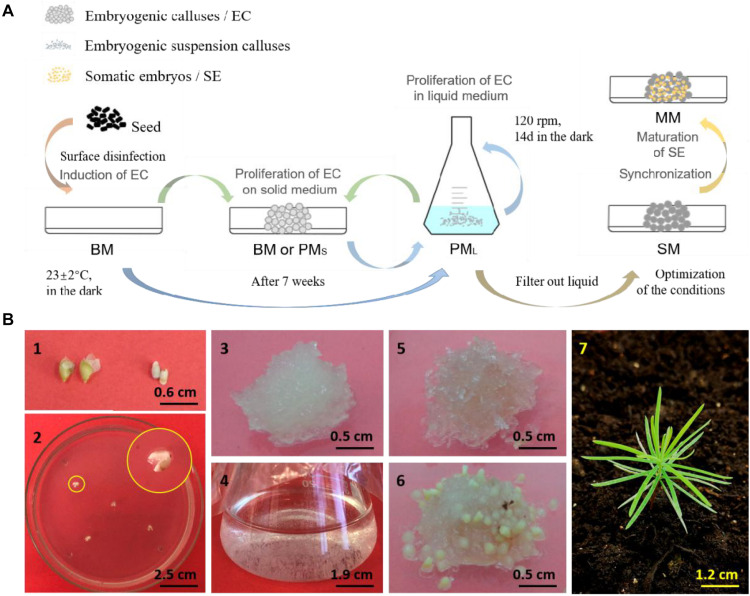
Somatic embryogenesis and regeneration of embryogenic callus of *L. olgensis*. The schematics **(A)** of somatic embryogenesis of *L. olgensis*. Representative images of the **(B)** immature seeds (1) exfoliated from megasporophyll and (2) inoculated into the basic medium (BM) to induce callus formation, (3) embryogenic callus (EC) cultured on proliferation medium (PM_S_) for 14 days, (4) EC cultured in liquid proliferation medium (PM_L_) for 7 days, (5) EC cultured on synchronization medium (SM) for 14 days, (6) induction of pro-embryo maturation on maturation medium (MM) for 15 days, and (7) plantlet from somatic embryo transplants.

The embryogenic suspension calluses were isolated from liquid medium and transferred to maturation medium (MM) consisting of the basic salts of BM supplemented with 20.0 mg⋅L^–1^ abscisic acid (ABA), 80.0 g⋅L^–1^ PEG_4_,_000_, 5.0 g⋅L^–1^ silver nitrate (AgNO_3_), and 60.0 g⋅L^–1^ sucrose. Somatic embryo maturation was induced for 7 weeks. Normal cotyledon embryo germination was observed after 7 days of inoculation on L&M woody plant basal medium (WPM; PhytoTech Labs, United States) supplemented with 20.0 g⋅L^–1^ sucrose, 4.0 g⋅L^–1^ agar, 3.0 g⋅L^–1^ vitamin B_1_ and 2.0 g⋅L^–1^ activated carbon under a 16 h/day photoperiod (50 μmol⋅m^–2^⋅s^–1^). To increase the efficiency of somatic embryogenesis and optimize the synchronization, embryonic calluses were cultured in synchronized culture medium (SM) before the somatic embryo maturation. To determine the optimum culture period, 0.2 g calluses were cultured on medium containing 1/4 BM basic salts with the addition of 60.0 g⋅L^–1^ sucrose, 10.0 g⋅L^–1^ inositol, 1.0 g⋅L^–1^ Gln, 0.5 g⋅L^–1^ CH, and 6.0 g⋅L^–1^ agar for 1, 7, 14, 21, or 28 days in the dark. In addition, different concentrations of sucrose (30, 60, and 90 g⋅L^–1^), inositol (1, 10, and 15 g⋅L^–1^), Gln (0, 0.5, and 1 g⋅L^–1^), CH (0, 0.25, and 0.5 g⋅L^–1^) and total, 1/2, or 1/4 BM salts were also tested for 14 days. The process is outlined in Figure 1A. Each treatment was performed in triplicate, and at least 15 embryogenic calluses of *L. olgensis* were examined for each experiment.

### Antibiotic Sensitivity Test

The embryogenic calluses were filtered out using a 60-mesh cellular sieve (0.3 mm), and the excess amount of water was absorbed on a sterile filter paper. The calluses were weighed, and 0.25 g was inoculated into 25 ml PM supplemented with 0, 100, 200, 300, or 400 mg⋅L^–1^ cefotaxime (Cef). After culturing for 14 days at 120 rpm and 23 ± 2°C in the dark, the proliferative calluses were separated as above and weighed, and 0.5 g calluses were seeded on solid PM (6 g⋅L^–1^ agar) supplemented with various concentrations (0, 10, 20, 30, or 40 mg⋅L^–1^) of kanamycin (Kan) or (0, 2, 4, 6, 8, or 10 mg⋅L^–1^) hygromycin (Hyg). The plates were incubated at 23 ± 2°C in the dark for 15 days. Each callus was weighed, transferred to SM (with the same respective concentration of Kan or Hyg), and cultured for 14 days and thereafter to antibiotic-supplemented MM. The number of somatic embryos was counted after 8 weeks. The experiment was repeated thrice, and at least 15 embryogenic calluses were examined each time.

### *Agrobacterium* Strain Cultivation and Transformation

*Agrobacterium tumefaciens* strain GV3101 was transformed with the pBI121, pCAMBIA 1300, and recombinant pCAMBIA-PTHCA 2-1 Promoter-GUS binary vectors (Supplementary Figure S1). The pBI121 contains the CaMV35S-activated β-glucuronidase (GUS) gene, the neomycin phosphotransferase gene (*npt II*) as selective markers, and Nos terminator. The plasmid pCAMBIA 1301 contains the hygromycin phosphotransferase gene (*hpt*) and the GUS gene under the control of CaMV35S. The modified p1300-*Pt*HCA 2-1 Promoter-GUS also contains the GUS gene under the control of the *P. trichocarpa PtHCA 2-1* promoter. The cryopreserved transformed bacteria were revived, and a single colony was spread on YEB solid medium containing 50 mg⋅L^–1^ hygromycin and 50 mg⋅L^–1^ rifampicin. After incubating the plates at 28°C for 2 days, the bacteria were inoculated into 25 ml YEP medium and cultured overnight at 28°C with constant shaking at 200 rpm till the logarithmic phase. The cells were harvested by centrifuging at 8,000 rpm for 15 min at 4°C and resuspended in liquid BM at the appropriate density.

### *Agrobacterium*-Mediated Transformation of Embryogenic Calluses

Freshly proliferated embryogenic calluses were harvested, and ∼5 g callus was inoculated into 20 ml *A. tumefaciens* suspension of OD_600_ 0.2, 0.4, 0.6, or 0.8 with/out 100 μM acetosyringone (AS). The calluses were infected for 15–20 min with periodic shaking to allow even dispersion, separated, dried on a sterile filter paper, and seeded into the PM. The callus bacteria were co-cultivated at 25°C in the dark for 1, 2, 3, or 4 days in the presence or absence of 100 μM AS.

The cultured calluses were washed sequentially with sterile water and 500 mg⋅L^–1^ Cef for 5 min each, dried on a sterilized filter paper, and cut into small pieces of diameter 1 ± 0.2 cm. The pieces were placed on PM supplemented with 200 mg⋅L^–1^ Cef and cultured at 25°C in the dark for 0, 3, 7, 14, or 21 days. The antibiotic-resistant calluses were selected thrice on PM containing 200 mg⋅L^–1^ Cef and 20 mg⋅L^–1^ Kan or 4 mg⋅L^–1^ Hyg at 25°C in the dark for 21 days, and the number of calluses was counted. All treatments were repeated at least three times, and at least 15 calluses were examined from each independent experiment.

### Molecular Analysis

Genomic DNA was isolated from the wild-type and transgenic calluses and somatic embryos using a plant DNA extraction kit (Qiagen, China) according to the manufacturer’s instructions. *GUS* and *npt II* genes were amplified by PCR using specific primers (Supplementary Table S3) and the Premix Taq DNA Polymerase Kit (Clontech, China) according to the manufacturer’s instructions. The PCR conditions were as follows: initial denaturation at 95°C for 3 min, 30 cycles at 94°C for 30 s, 60°C for 30 s, and 72°C for 45 s and a final extension step at 72°C for 10 min. For gene expression analysis, total RNA was extracted from the calluses and somatic embryos using the RNeasy Plant Mini Kit (Qiagen, China), reverse transcribed using the ReverTra Ace kit (Clontech, China) according to the manufacturer’s instructions, and amplified by reverse transcription (RT)-PCR using the same primers as listed in Supplementary Table S3.

### GUS Staining

Glucuronidase staining was performed as described previously (Jefferson et al., 1987). Briefly, hygromycin-resistant callus and somatic embryos were immersed in a solution consisting of 2 mM X-Gluc (5-bromo-4-chloro-3-indolyl-β-D-glucuronide), 5 mM ferro-ferricyanide buffer, 100 mM Na-phosphate buffer (pH 7.0), 10 mM EDTA, and 0.1% Triton X-100 and incubated at 37°C for 7 days. The resistant embryogenic calluses stained blue were counted under a microscope, and the staining intensity was evaluated by extracting the chlorophyll of somatic embryos using 75% (v/v) ethanol.

### Statistical Analysis

SPSS 18.0 (Chicago, United States) was used for all data analyses. Data were compared by ANOVA, followed by Fisher’s LSD test, and *p* < 0.05 was considered statistically significant.

## Results

### Synchronization of *L. olgensis* Calluses Improved Embryonic Maturation

After 7 days of synchronized culture, the embryogenic calluses surface changed from translucent to opalescent, and the internal areas were browned 28 days later. Following synchronization, the calluses were exposed to maturation stimuli for approximately 7 days. The pro-embryo masses sprouted short, milky-yellow rod-like protuberances on the surface, which is indicative of close to maturation. After 15 days, some embryos differentiated and formed cotyledons (Figure 1B). In contrast, the unsynchronized calluses began to mature approximately 45 days after the initial ABA stimulus.

**FIGURE 2:**
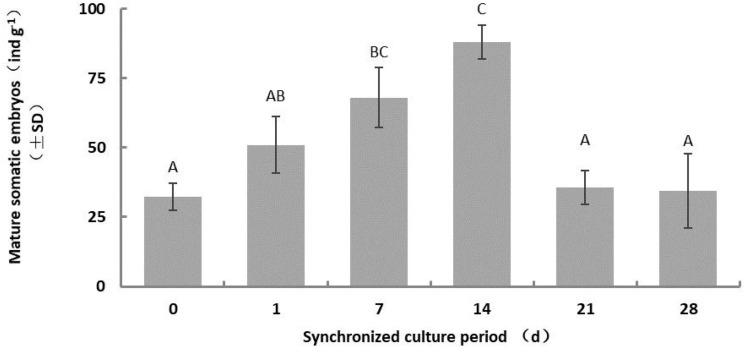
Effect of the synchronous culture period on somatic embryo maturation of *L. olgensis*. SM contained 1/2 BM salts, 60 g⋅L^–1^ sucrose, 10 g⋅L^–1^ inositol, 1 g⋅L^–1^ glutamine, and 0.5 g⋅L^–1^ hydrolyzed casein. Each value represents the mean of three independent experiments with standard deviation (SD). Different letters indicate *p* < 0.05 (Duncan’s multiple range test).

**FIGURE 3:**
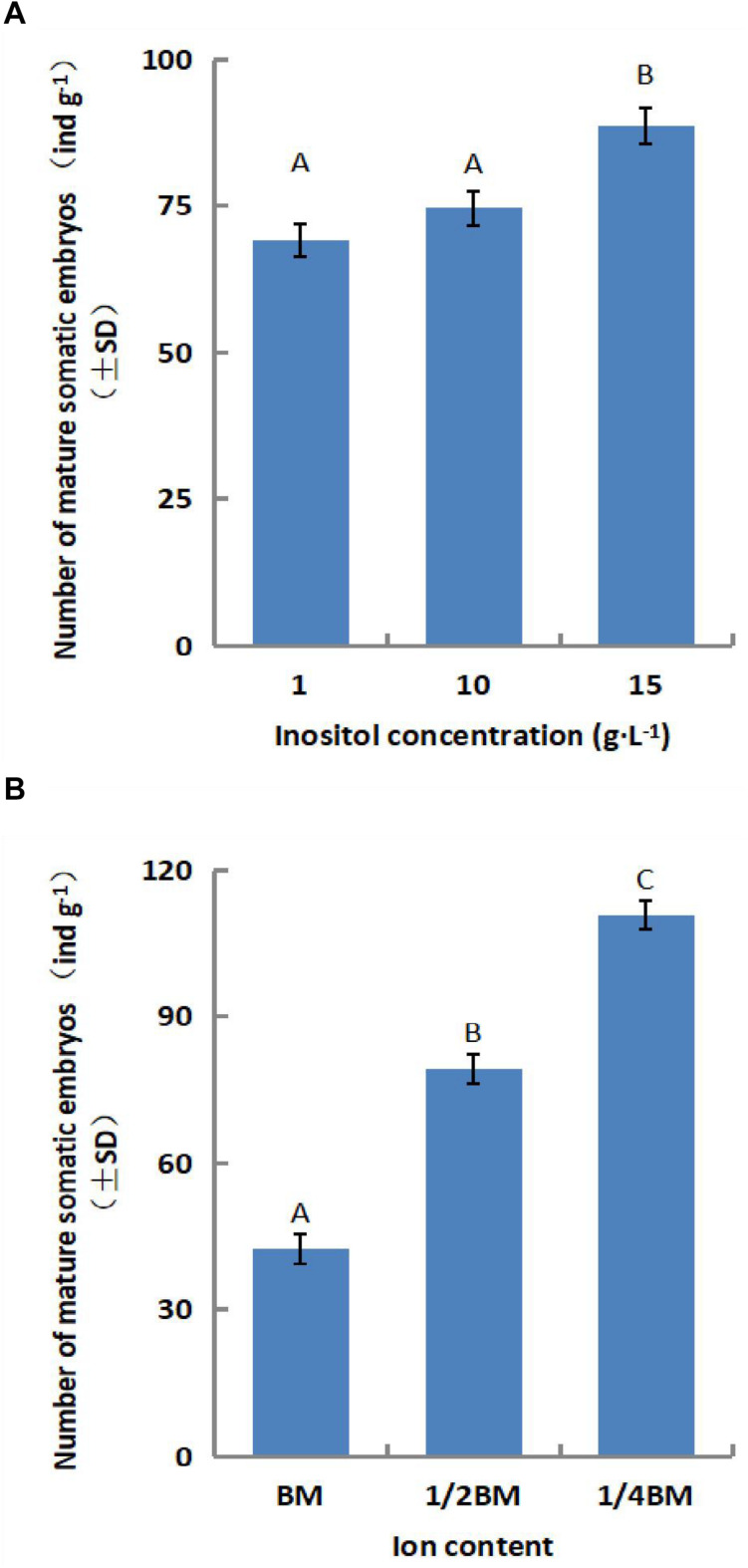
Effect of the SM composition on somatic embryo maturation of *L. olgensis.* The effects of **(A)** inositol concentration and **(B)** ion content on the number of somatic embryos. SM contained 1/4 BM salts, 10 g⋅L^–1^ inositol, 1 g⋅L^–1^ glutamine, 0.5 g⋅L^–1^ hydrolyzed casein, and 60 g⋅L^–1^ sucrose. Each value represents the mean of three independent experiments with standard deviation (SD). Different letters indicate *p* < 0.05 (Duncan’s multiple range test).

The number of somatic embryos increased gradually for a short period during synchronous culture (day 1–14), peaked on the 14th day, and decreased after the 21st day (Figure 2). Therefore, we limited the duration of synchronized culture to 14 days for the subsequent experiments. The yield of mature somatic embryos was also affected by the concentration of salts ion (*p* = 0.000) and inositol (*p* = 0.002) in the synchronization medium (Figures 3A,B). Somatic embryogenesis increased by 160.76% when the salts ion concentration decreased to 25%, whereas the addition of 15 g⋅L^–1^ inositol increased the average embryo yield to 88.77/g. However, the concentration of sucrose, Gln, and CH did not significantly affect the number of somatic embryos, although the combination of 60 g⋅L^–1^ sucrose, 0.5 g⋅L^–1^ Gln, and 0.25 g⋅L^–1^ CH promoted subsequent germination (data not shown). Taken together, these results indicate that synchronization of *L. olgensis* embryogenic calluses can accelerate somatic embryo maturation.

### Effects of Antibiotics on Callus Proliferation and Somatic Embryogenesis

Cefotaxime inhibited the proliferation of *L. olgensis* embryogenic calluses at concentrations exceeding 200 mg⋅L^–1^ (Supplementary Figure S2A), whereas kanamycin exerted a significant inhibitory effect at 10 mg⋅L^–1^ and altogether stalled callus proliferation at 20 mg⋅L^–1^ (Supplementary Figure S2B). In addition, 10 mg⋅L^–1^ kanamycin significantly inhibited the maturation of somatic embryos. The *L. olgensis* calluses were highly sensitive to hygromycin. Compared with the control, 2 mg⋅L^–1^ Hyg decreased the multiplication of embryogenic calluses by 65.66% (Supplementary Figure S2C) and completely inhibited embryo maturation, whereas 4 mg⋅L^–1^ of the antibiotic almost stalled embryogenic callus proliferation. Variance analysis showed that Cef (*p* = 0.000), Kan (*p* = 0.000), and Hyg (*p* = 0.000) significantly influenced the growth of embryonic callus, and Kan (*p* = 0.000) and Hyg (*p* = 0.000) affected somatic embryogenesis.

### *Agrobacterium* Load and Co-Culture Duration Affected Transformation Efficiency

**FIGURE 4:**
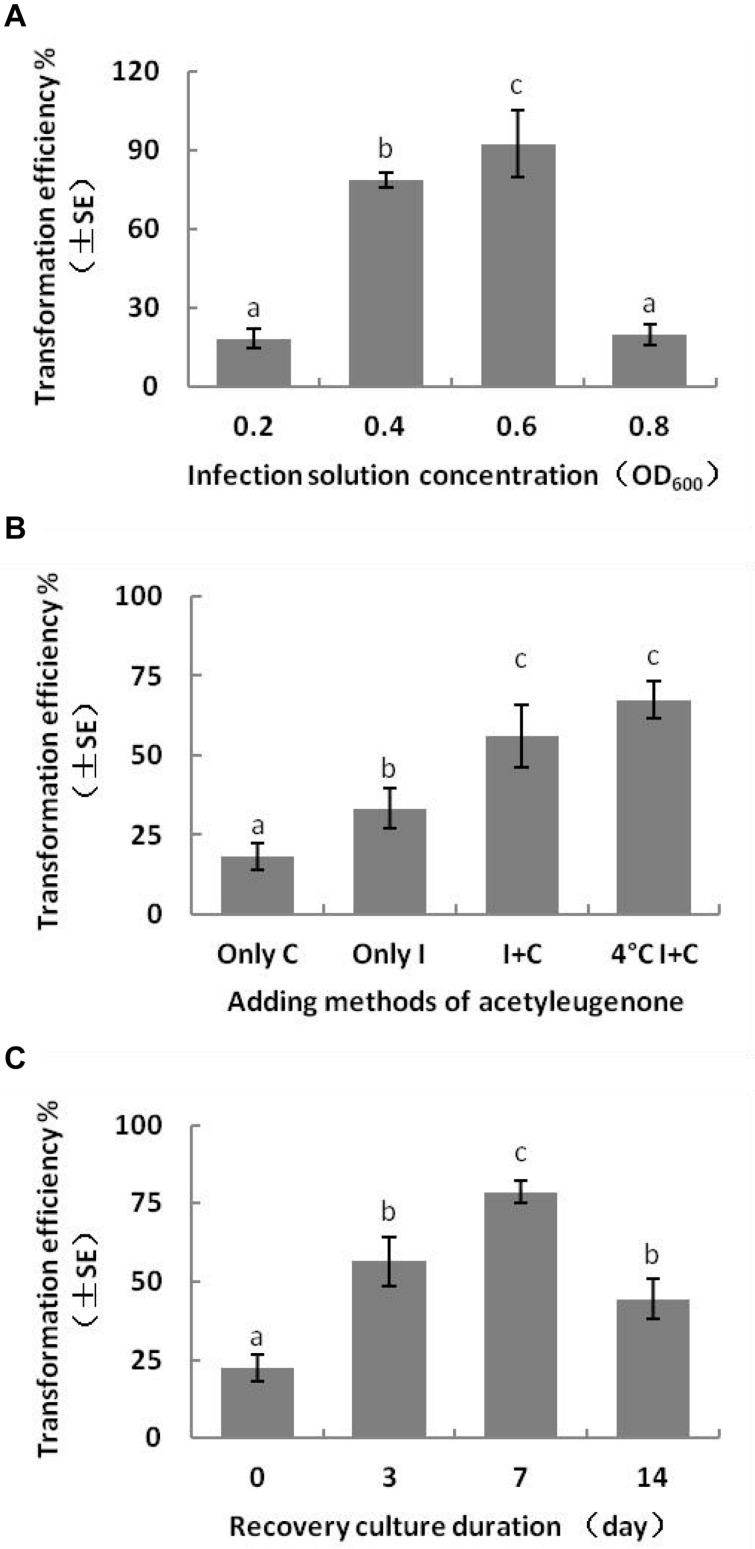
Effect of genetic transformation conditions on transformation efficiency of *L. olgensis*. Effects of **(A)** *Agrobacterium* load, **(B)** presence of acetosyringone in different stages, and **(C)** duration of the recovery period after disinfection of callus with cefotaxime. Acetosyringone (100 μM) was added in the bacterial suspension and/or co-culture medium or pre-chilled for 1 h. The resistant calluses were selected on the screening medium containing 4 mg⋅L^–1^ hygromycin. Each value represents the mean of three independent experiments with the standard error (SE). Different letters indicate *p* < 0.05 (Duncan’s multiple range test).

The transformation efficiency of embryogenic calluses was the highest at 92.5 ± 22.13% when infected with *Agrobacterium* suspension of OD_600_ 0.6 and decreased slightly with a lower bacterial load (OD_600_ 0.4). In contrast, low (OD_600_ 0.2) and high (OD_600_ 0.8) density bacterial suspensions decreased the transformation efficiency. Previous reports show that T-DNA transfer and integration from *A. tumefaciens* to the recipient plant cells require at least 16 h. Consistent with this, the transformation efficiency of the embryogenic calluses was the highest after 3 days of bacterial co-culture, and Kan-resistant calluses were not formed when the bacteria were removed. Extensive bacterial overgrowth was observed with highly dense *Agrobacterium* suspension (OD_600_ > 0.8) and co-culture duration longer than 4 days. The multiple sterilization steps required to remove the bacteria led to considerable loss of the transformed embryogenic calluses. Therefore, to ensure maximum transformation efficiency and transformant recovery, we infected the calluses with *Agrobacterium* suspension of OD_600_ 0.6 for 15–20 min and co-cultured them for 3 days (Figure 4A). Directional transfer of T-DNA from *A. tumefaciens* to target cells requires the expression of the *Vir* genes (Turk et al., 1993; Wang et al., 2002), which can be activated by some carbohydrates and phenols (Song et al., 1991). The average transformation efficiency of embryonic calluses reached 56.11 ± 16.95% when 100 μM AS was present in both the infection solution and co-culture medium, whereas the optimizing effect was not observed when only either of the two were supplemented with AS. In addition, the efficiency of transformation also increased when the bacterial suspension was chilled at 4°C for 1 h before infection (Figure 4B).

### Effect of Degerming and Screening Methods on Transformation Efficiency

**FIGURE 5:**
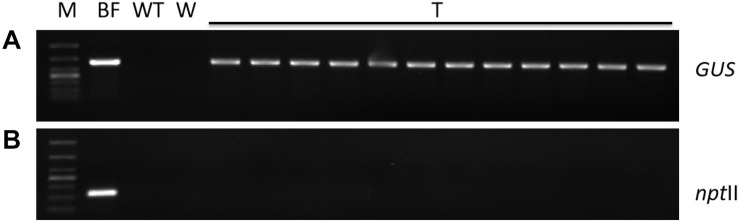
Molecular detection of transgenic embryogenic callus of *L. olgensis.* PCR amplification of **(A)** GUS and **(B)** npt II gene in *A. tumefaciens* GV3101 containing pCAMBIA1301, untransformed wild-type (WT), and hygromycin-resistant callus (T). Lanes from left to right: 1 kb plus DNA ladder, bacterial fluid, WT callus DNA, water control, and resistant callus DNA (T_1__–__12_).

**FIGURE 6:**
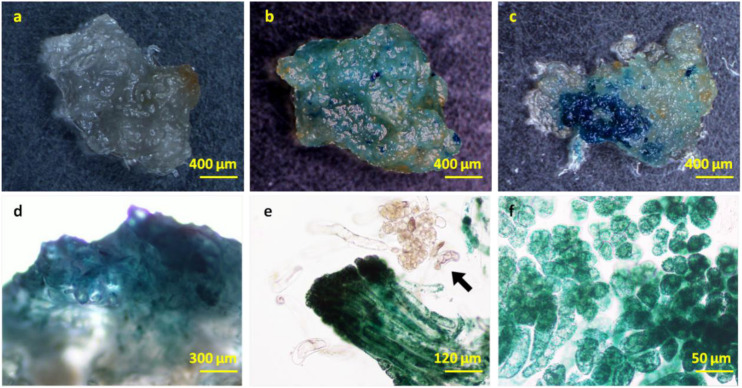
GUS staining in the transgenic *L. olgensis* embryogenic callus. GUS staining of **(a)** WT, **(b)** kanamycin-, and **(c)** hygromycin-resistant calluses infected with *A. tumefaciens* GV3101 containing plasmid pBI121 and pCAMBIA1301, respectively. Expression pattern of 35S promoter-driven GUS in embryogenic callus **(d)**, whole embryogenic suspended mass **(e)**, and embryogenic cells **(f)**. The arrow indicates a chimeric cell mass lacking exogenous GUS gene activity.

In order to ensure normal growth of the *Agrobacterium*-transformed plant tissues, the latter are treated with bacteriostatic antibiotics after co-culture to check further bacterial growth (Kumlehn et al., 2006). The bacterial growth on calluses was completely inhibited after washing the latter for 10 min with 100 mg⋅L^–1^ Cef (Supplementary Figure S3) or for 5 min with 200 mg⋅L^–1^ Cef. Furthermore, two successive washes with 200 mg⋅L^–1^ Cef prevented any secondary contamination of *A. tumefaciens*. However, the transformation efficiency was adversely affected when the tissues were inoculated in the screening medium right after bacteriostatic antibiotic treatment, and the average number of resistant tissues formed per slice (approximately 1.4 ± 0.1 cm^2^) was only 0.23 ± 0.08 (Figure 4C). A recovery period of 3 or 7 days post-disinfection in PM lacking the screening antibiotics increased the number of resistant calluses to 0.56 ± 0.13 and 0.79 ± 0.60 per slice, respectively. In contrast, a longer recovery period of 14 days decreased the transformation efficiency to 44.44 ± 11.11% (Table 1) due to excessive reproduction of the pseudo-positive tissues that competed for nutrients with the transformed tissues. Furthermore, the false positive rate of the resistant tissues was nearly 100% after 21 days. The embryogenic calluses of *L. olgensis* were more sensitive to Hyg than to Kan, indicating a possible effect of antibiotic screening on transformation efficiency. Indeed, the false positive rate of the resistant tissues was ∼86.71% when screened with 20 mg⋅L^–1^ Kan compared with only 11.66% with 4 mg⋅L^–1^ Hyg (Table 2). Therefore, Hyg was more beneficial for screening the genetically transformed calluses of *L. olgensis*. The transgenic calluses were further verified by analyzing the expression of *GUS* and *npt II* genes. The negative and positive controls were the uninfected tissues and suspension of *Agrobacterium* GV3101 transformed with binary vector, respectively. As shown in Figures 5A,B, 6A–F, GUS was amplified in the transgenic and not in the wild-type calluses. In addition, *npt* II was not detected in any of the transformed tissues, indicating the absence of *Agrobacterium* contamination.

**TABLE 1:** Effects of the duration of recovery post-bacteriostasis on the transformation efficiency of *L. olgensis*.

Recovery culture period (days)	Number of resistant callus*	GUS stained rate of resistant callus (%)**	Positive rate of PCR detection (%)***
0	10/45	10.00 (±14.14)^b^	55.00 (±7.07)^b^
3	25/45	31.11 (±10.18)^a^	91.07 (±7.78)^a^
7	34/45	32.14 (± 9.45)^a^	88.89 (±11.11)^a^
14	20/45	15.48 (±1.68)^ab^	44.44 (±9.62)^b^
21	22/45	00.00 (±0.00)^c^	00.00 (±0.00)^c^

Each value represents the mean of three independent experiments with the standard deviation (SD). Approximately 15 embryogenic calluses of Larix olgensis were examined for each individual experiment. Values with different letters are significantly different at p < 0.05 (Duncan’s multiple range test). * The number of resistant calluses obtained on the screening medium containing 4 mg⋅L^–1^ hygromycin from 45 pieces of embryogenic cell mass for subsequent detection. ** Corresponds to the frequency of resistant calluses that gave rise to GUS staining. *** The PCR positive detection frequency of resistant calluses was determined.

**TABLE 2:** Effects of kanamycin and hygromycin on the transformation efficiency of *L. olgensis.*

Antibiotics for screening	Number of resistant callus*	GUS stained rate of resistant callus (%) **	Positive rate of PCR detection (%) ***
Kanamycin	37/45	13.69 (±1.03)	13.29 (±12.54)
Hygromycin	44/45	25.93 (±6.42)	89.34 (±10.72)

Each value represents the mean of three independent experiments with the standard deviation (SD). Approximately 15 embryogenic calluses of Larix olgensis were examined for each individual experiment. * The number of resistant calluses obtained on the screening medium was counted. The concentrations of kanamycin and hygromycin in the screening medium were 20 and 4 mg⋅L^–1^, respectively. ** Corresponds to the frequency of resistant calluses that gave rise to GUS staining. *** The PCR positive detection frequency of resistant calluses was determined.

### Transformed Buds Expressed GUS in a Tissue-Specific Manner Under the Control of *PtHCA 2-1* Promoter

To further validate the applicability of somatic embryogenesis, we expressed the GUS gene under the control of the *P. trichocarpa PtHCA 2-1* promoter in embryogenic calluses and somatic embryo seedlings. The detailed flowchart of transformation is shown in Supplementary Figure S4. *HCA2* (high cambial activity 2) regulates interfascicular cambium formation and vascular tissue development in *Arabidopsis* (Pineau et al., 2005). It is constitutively expressed in the vascular tissues from the seedling stage to the mature plant (Pineau et al., 2005; Guo et al., 2009). Premature and many cambial cell divisions in both the fascicular and interfascicular regions typically result in the loss of the alternate vascular bundle/fiber organization. *Arabidopsis* strains expressing mutant HCA show increased vascular tissue development, stunting, and a delay in flowering and senescence (Guo et al., 2009).

**FIGURE 7:**
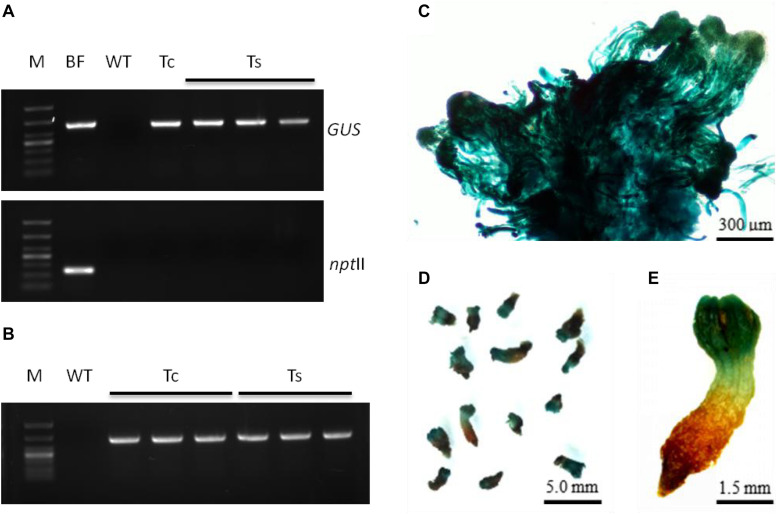
Molecular detection and expression analysis of transgenic embryogenic cultures of *L. olgensis* with GUS gene. **(A)** PCR amplification of GUS and *npt* II genes in *Agrobacterium* GV3101 containing p1300-*Pt*HCA 2-1pro-GUS, untransformed wild-type (WT), and transgenic callus (Tc) or somatic embryos (Ts). Lane M is 1 kb ladder. **(B)** RT-PCR amplification of GUS gene in WT and transgenic embryogenic cultures (Tc or Ts). *Pt*HCA 2-1pro-GUS transformed callus **(C)** or somatic embryos **(D,E)** after staining with X-Gluc.

In our study, the transgenic calluses, somatic embryos, and seedling terminal buds stained an intense blue color with X-Gluc, indicating GUS expression (Figure 7A–E). Interestingly, the resistant callus expressed GUS in all cells, including the embryos and suspensor without any visible chimera. However, the seedling roots arising from the germination of these resistant tissues did not express GUS, indicating that the *PtHCA 2-1* promoter ensures tissue-specific transcription of exogenous genes in *L. olgensis*. Furthermore, the transgenic and wild-type calluses yielded similar number of somatic embryos (Supplementary Table S4), indicating that genetic transformation and expression of exogenous genes do not affect the maturation and germination of somatic embryos. The transgenic seedlings were transplanted into soil mixed with vermiculite (3:1), and their survival rate after 4 weeks was higher than 95% (Supplementary Figure S5).

## Discussion

Plant somatic embryogenesis is a highly complex process (Sharifi et al., 2012; Smertenko and Bozhkov, 2014) and requires highly calibrated conditions for *ex vivo* culture. Embryonic development of pine cones can be divided into the pro-embryo, early embryogenesis, and late embryogenesis stages (Smertenko and Bozhkov, 2014). Morphologically, the pro-embryo mass period can be further divided into stages I, II, and III (Filonova et al., 2000b), of which only stage III can further develop into mature somatic embryos. Cytokinins, such as 6-BA and KT, and auxins, such as 2,4-D and naphthaleneacetic acid (NAA), added to promote plant tissue growth *in vitro* can skew cell division and polarity of the pro-embryos, resulting in their disintegration before stage III (Nishl et al., 1997; Filonova et al., 2000a). Therefore, we synchronized the embryogenic calluses of *L. olgensis* by removing growth regulators from the medium at an early stage, which significantly accelerated somatic embryo maturation. Interestingly, the number of somatic embryos was affected by the ion and inositol content in the synchronized culture medium, and this relatively novel molecular mechanism needs further study. In addition, although glutamine or hydrolyzed casein alone had no significant effect on the embryo yield, their combined effect on somatic embryogenesis was significant. The major factors affecting *Agrobacterium*-mediated transformation of conifers are the physiological status of the plant recipient, the bacterial strain and plasmid vectors, and the infection and screening conditions (Wu et al., 2015; Kim et al., 2016; Aggarwal et al., 2018; Li et al., 2018). Furthermore, *Agrobacterium* load and the infection time are also important determinants for successful transformation (Li et al., 2017, 2018). The optimum transformation efficiency in this study was obtained with a moderate bacterial load and co-culturing for 2–3 days, which is similar to previous findings (Wang, 2007; Zhu et al., 2011). A sufficient co-culture duration of 2–3 days is necessary for ensuring complete transfer of the T-DNA from *A. tumefaciens* to plant cells. Excessive *Agrobacterium* load and/or longer infection time can considerably lower plant viability and reduce the transformation efficiency. In addition, phenolic substances secreted by damaged plant cells attract *A. tumefaciens via* chemotaxis and promote the transfer and integration of T-DNA by inducing the *Vir* genes. Previous studies have shown that acetosyringone can improve the transformation efficiency of conifers (Song et al., 1991; Zhu et al., 2011). Although the addition of inducers does not necessarily increase the efficiency of *Agrobacterium*-mediated transformation, and some may even have a negative effect, we found that acetosyringone increased the transformation rate of *L. olgensis*.

Optimal selection pressure, usually an antibiotic, is another determinant of the efficacy of genetic transformation of conifers (Wu et al., 2015; Li et al., 2017); therefore, it is essential to determine the antibiotic sensitivity of the recipient in order to improve transformation efficiency, allow normal growth of the transformants, and reduce the false positive rate. *NptII* (neomycin phosphotransferase gene), *hpt* (hygromycin phosphotransferase gene), and *bar* (glyphosate acetate transferase gene) are commonly used as selection markers for conifer genetic transformation (Duchesne et al., 1993; Wenck et al., 1999; Tang et al., 2001). We found that the embryogenic callus of *L. olgensis* was highly sensitive to all antibiotics, and 4 mg⋅L^–1^ Hyg was selected for screening transformants due to the lower false positive rate.

Taken together, *Agrobacterium*-mediated transformation of synchronized embryonic calluses can rapidly produce a relatively large number of transgenic somatic embryos or plants and can also be scaled up. Compared with the previous studies on larches (Supplementary Table S1), *L. olgensis* was genetically transformed at significantly higher efficiency under appropriate conditions. This approach not only can accelerate the generation of genetically superior *L. olgensis* varieties but can also potentially aid protein subcellular localization studies and other applications requiring transgene expression. Our future efforts will focus on further enhancing the transformation efficiency of *L. olgensis* and conducting functional gene studies.

## Conclusion

We improved the protocol of larch somatic embryogenesis and developed a rapid and simple transformation method for *L. olgensis* embryogenic calluses using *A. tumefaciens* strain GV3101 expressing pCAMBIA 1300, bacterial density OD_600_ of 0.6, 3 days of co-culture, 100 μM acetosyringone as inducer, and transformant screening with 4 mg⋅L^–1^ Hyg. Under these optimum conditions, the transformation efficiency was almost 90%, which is higher compared with that reported for other conifers and larch species. This novel method can potentially accelerate genetic improvement of *L. olgensis*.

## Data Availability Statement

The original contributions presented in the study are included in the article/Supplementary Material, further inquiries can be directed to the corresponding author/s.

## Author Contributions

SL and HZ conceived the study. YS and SL designed the study. YS, XB, and NW performed the experiments. YS, SD, YY, and HD contributed to data interpretation. YS and SL wrote the manuscript. All authors read and approved the final manuscript.

## Conflict of Interest

The authors declare that the research was conducted in the absence of any commercial or financial relationships that could be construed as a potential conflict of interest.

## Supplementary Material

Supplementary Figure 1 Schematic representation of binary expression vectors used in this study.

Supplementary Figure 2 Effect of antibiotics on the proliferation of embryogenic callus of *L. olgensis*.

Supplementary Figure 3 Comparison of the bacteriostatic effects of different concentrations of cefotaxime.

Supplementary Figure 4 Schematic representation of the timeline for *A. tumefaciens*-mediated transformation of *L. olgensis* embryogenic callus.

Supplementary Figure 5 The seedling transplanting of transgenic *L. olgensis.*

Supplementary Table 1 Efficiency and conditions of *Agrobacterium-*mediated genetic transformation of *Larix.*

Supplementary Table 2 Composition of BM (basic medium).

Supplementary Table 3 List of primers used for PCR.

Supplementary Table 4 Comparison of the number of mature somatic embryos and somatic embryo germination rate of transgenic and non-transgenic embryogenic callus of *L. olgensis.*

## References

[B1] Aggarwal P. R. Nag P. Choudhary P. Chakraborty N. Chakraborty S. (2018). Genotype-independent *Agrobacterium rhizogenes*-mediated root transformation of Chickpea: a rapid and efficient method for reverse genetics studies. *Plant Methods* 14:55. 10.1186/s13007-018-0315-6 29988950 PMC6034309

[B2] Belide S. Vanhercke T. Petrie J. R. Singh S. P. (2017). Robust genetic transformation of Sorghum (*Sorghum bicolor* L.) using differentiating embryogenic callus induced from immature embryos. *Plant Methods* 13:109. 10.1186/s13007-017-0260-9 29234458 PMC5723044

[B3] Boruszewski P. Jankowska A. Kurowska A. (2017). Comparison of the structure of juvenile and mature wood of *Larix decidua* mill. from fast-growing plantations in Poland. *Bioresources* 12 1813–1825. 10.15376/biores.12.1.1813-1825

[B4] Charest P. J. Yvonne D. Christine W. Catherine J. Ulriche S. Klimaszewska K. (2011). Transient expession of foreign chimeric genes in the gymnosperm hybrid larch following electroporation. *Can. J. Bot.* 69 1731–1736. 10.1139/b91-220

[B5] Duchesne L. Lelu M. A. Aderkas V. P. Charest P. J. (1993). Microprojection-mediated DNA delivery in embryogenic cells of *Larix* spp. *Can. J. Forest Res.* 23 312–316. 10.1139/x93-042

[B6] Filonova L. H. Bozhkov P. V. Arnold S. V. (2000a). Developmental pathway of somatic embryogenesis in *Picea abies* as revealed by time-lapse tracking. *J. Exp. Bot.* 51 249–264. 10.1093/jexbot/51.343.249 10938831

[B7] Filonova L. H. Bozhkov P. V. Brukhin V. B. Daniel G. Zhivotovsky B. Von A. S. (2000b). Two waves of programmed cell death occur during formation and development of somatic embryos in the gymnosperm, Norway spruce. *J. Cell Sci.* 113(Pt 24), 4399–4411. 10.1007/BF02703793 11082033

[B8] Flachowsky H. Haaanke M. V. Peil A. Strauss S. H. Fladung M. (2009). A review on transgenic approaches to accelerate breeding of woody plants. *Plant Breed.* 128 217–226. 10.1111/j.1439-0523.2008.01591.x

[B9] Guo Y. Qin G. Gu H. Qua L. J. (2009). Dof5.6*/HCA*2, a Dof transcription factor gene, regulates interfascicular cambium formation and vascular tissue development in *Arabidopsis*. *Plant Cell* 21 3518–3534. 10.1105/tpc.108.064139 19915089 PMC2798324

[B10] Han S. Wang Q. Zhou C. Liu G. Qi L. (2006). Transformation of hybrid larch (*Larix leptolepis* × *L. olgensis) with P*5CS gene cloned from *Populus simonii* cDNA library. *Biotechnol. Bull.* 32 88–92.

[B11] Hu X. Yang J. Li C. (2015). Transcriptomic response to nitric oxide treatment in *Larix olgensis* Henry. *Int. J. Mol. Sci.* 16 28582–28597. 10.3390/ijms1612 26117 26633380 PMC4691064

[B12] Huang Y. Diner A. M. Karnosky D. F. (1991). Agrobacterium rhizogenes-mediated genetic transformation and regeneration of a conifer: *Larix decidua*. *In Vitro Cell. Dev. Biol. Plant* 27 201–207. 10.1007/BF02632217

[B13] Ismail G. Schnabl H. Zoglauer K. Boehm R. (2004). *Agrobacterium*-mediated transformation of *Larix decidua*: an assessment of factors influencing the efficiency of gus gene transfer. *J. Appl. Bot. Food Qual.* 78 83–90.

[B14] Jefferson R. A. Bevan M. Kavanagh T. (1987). The use of the *Escherichia coli* beta-glucuronidase as a gene fusion marker for studies of gene expression in higher plants. *Biochem. Soc. Trans.* 15 17–18. 10.1042/bst0150017 3549385

[B15] Keith C. T. Chauret G. (2011). Basic wood properties of European larch from fast-growth plantations in Eastern Canada. *Can. J. Forest Res.* 18 1325–1331. 10.1139/x88-204

[B16] Kim M. J. An D. J. Moon K. B. Cho H. S. Min S. R. Sohn J. H. (2016). Highly efficient plant regeneration and *Agrobacterium*-mediated transformation of *Helianthus tuberosus* L. *Ind. Crops Prod.* 83 670–679. 10.1016/j.indcrop.2015.12.054

[B17] Klimaszewska K. Devantier Y. Lachance D. Lelu M. A. Charest P. J. (1997). *Larix laricina* (tamarack): somatic embryogenesis and genetic transformation. *Can. J. Forest Res.* 27 538–550. 10.1139/x96-208

[B18] Kumlehn J. Serazetdinova L. Hensel G. Becker D. Loerz H. (2006). Genetic transformation of barley (*Hordeum vulgare* L.) via infection of androgenetic pollen cultures with *Agrobacterium tumefaciens*. *Plant Biotechnol. J.* 4 251–261. 10.1111/j.1467-7652.2005.00178.x 17177801

[B19] Levee V. Garin E. Klimaszewska K. Seguin A. (1999). Stable genetic transformation of White Pine (*Pinus strobus* L.) after cocultivation of embryogenic tissues with *Agrobacterium tumefaciens*. *Mol. Breed.* 5 429–440. 10.1023/A:1009683605841

[B20] Levee V. Lelu M. A. Jouanin L. Cornu D. Pilate G. (1997). *Agrobacterium tumefaciens*-mediated transformation of Hybrid Larch (*Larix kaempferi* × *L.decidua*) and transgenic plant regeneration. *Plant Cell Rep.* 16 680–685. 10.1007/s002990050301 30727618

[B21] Li H. P. Li K. Guo Y. T. Guo J. G. Miao K. T. Botella J. R. (2018). A transient transformation system for gene characterization in upland cotton (*Gossypium hirsutum*). *Plant Methods* 14:50. 10.1186/s13007-018-0319-2 29977323 PMC6013946

[B22] Li S. Zhen C. Xu W. Wang C. Cheng Y. (2017). Simple, rapid and efficient transformation of genotype Nisqually-1: a basic tool for the first sequenced model tree. *Sci. Rep.* 7:2638. 10.1038/s41598-017-02651-x 28572673 PMC5453977

[B23] Lin X. F. Zhang W. B. Takechi K. Takio S. Ono K. Takano H. (2005). Stable genetic transformation of *Larix gmelinii L.* by particle bombardment of zygotic embryos. *Plant Cell Rep.* 24 418–425. 10.1007/s00299-005-0955-7 15830196

[B24] Nishl A. Kato K. Takahashi M. Yoshida R. (1997). Partial synchronization of carrot cell culture by auxin deprivation. *Physiol. Plant* 39 9–12. 10.1111/j.1399-3054.1977.tb09277.x

[B25] Pineau C. Freydier A. Ranocha P. Jauneau A. Turner S. Lemonnier G. (2005). Hca: an *Arabidopsis* mutant exhibiting unusual cambial activity and altered vascular patterning. *Plant J.* 44 271–289. 10.1111/j.1365-313X.2005.02526.x 16212606

[B26] Prakash M. G. Gurumurthi K. (2009). Genetic transformation and regeneration of transgenic plants from precultured cotyledon and hypocotyl explants of *Eucalyptus tereticornis* Sm. Using *Agrobacterium tumefaciens*. *In Vitro Cell. Dev. Biol. Plant* 45 429–434. 10.1007/s11627-008-9179-1

[B27] Qi L. W. Han Y. F. Li L. Ewald D. Han S. Y. (2000). The somatic embryogenesis and establishment of transformation experiment system in *Larix principis-Rupprechtii*. *Acta Biol. Exp. Sin.* 33 357–365. 12549075

[B28] Ratjens S. Mortensen S. Kumpf A. Bartsch M. Winkelmann T. (2018). Embryogenic callus as target for efficient transformation of cyclamen persicum enabling gene function studies. *Front. Plant Sci.* 9:1035. 10.3389/fpls.2018.01035 30087683 PMC6066641

[B29] Ren Y. C. Zhang J. Liang H. Y. Wang J. M. Yang M. S. (2017). Inheritance and expression stability of exogenous genes in insect-resistant transgenic poplar. *Plant Cell Tissue Organ Cult.* 130 567–576. 10.1007/s11240-017-1247-y

[B30] Sharifi G. Ebrahimzadeh H. Ghareyazie B. Gharechah J. Vatankhah E. (2012). Identification of differentially accumulated proteins associated with embryogenic and non-embryogenic calli in Saffron (*Crocus sativus* L.). *Proteome Sci.* 10:3. 10.1186/1477-5956-10-3 22243837 PMC3349542

[B31] Smertenko A. Bozhkov P. V. (2014). Somatic embryogenesis: life and death processes during apical-basal patterning. *J. Exp. Bot.* 65 1343–1360. 10.1093/jxb/eru005 24622953

[B32] Song Y. Zhang H. Li S. Li S. (2016). Relationship between the induction of embryogenic callus of larch and the morphology of immature embryos. *J. North-East Forest. Univ.* 44 25–30.

[B33] Song Y. N. Shibuya M. Ebizuka Y. Sankawa U. (1991). Synergistic action of phenolic signal compounds and carbohydrates in the induction of virulence gene expression of *Agrobacterium tumefaciens*. *Chem. Pharmaceut. Bull.* 39 2613–2616. 10.1248/cpb.39.2613 1806280

[B34] Takata N. Eriksson M. E. (2012). A simple and efficient transient transformation for hybrid Aspen (*Populus tremula* × *P. tremuloides)*. *Plant Methods* 8:30. 10.1186/1746-4811-8-30 22871142 PMC3476444

[B35] Tang W. Sederoff R. Whetten R. (2001). Regeneration of transgenic Loblolly pine (*Pinus taeda* L.) from Zygotic Embryos Transformed with *Agrobacterium tumefaciens*. *Planta* 213 981–989. 10.1007/s004250100566 11722135

[B36] Turk S. C. Lange R. P. Sonneveld E. Hooykaas P. J. (1993). The chimeric VirA-tar receptor protein is locked into a highly responsive state. *J. Bacteriol.* 175 5706–5709. 10.1128/JB.175.17.5706-5709.1993 8366057 PMC206631

[B37] Vain P. Harvey A. Worland B. Ross S. Snape J. W. Lonsdale D. (2004). The effect of additional virulence genes on transformation efficiency, transgene integration and expression in rice plants using the pGreen/pSoup dual binary vector system. *Transgenic Res.* 13 593–603. 10.1007/s11248-004-2808-5 15672840

[B38] Wang J. (2007). *Studies on the Transformation of Larix Based the System of Somatic Embryogenesis.* Dissertation for the Degree Beijing: Chinese Academy of Forestry.

[B39] Wang W. Li C. Yang J. Zhang H. Zhang S. (2009). Somatic embryogenesis and plantlet regeneration from immature zygotic embryos of Hybrid Larch. *Sci. Silvae Sin.* 45 34–38.

[B40] Wang Y. Gao R. Lynn D. G. (2002). Ratcheting Up vir gene expression in *Agrobacterium tumefaciens*: coiled coils in histidine kinase signal transduction. *Chembiochem* 3 311–317. 10.1002/1439-7633 11933231

[B41] Wenck A. R. Quinn M. Whetten R. W. Pullman G. Sederoff R. (1999). High-efficiency *Agrobacterium*-mediated transformation of Norway spruce (*Picea abies*) and Loblolly Pine (*Pinus taeda*). *Plant Mol. Biol.* 39 407–416. 10.1023/A:1006126609534 10092170

[B42] Wu J. Liu C. Seng S. S. Khan M. A. Sui J. J. Gong B. H. (2015). Somatic Embryogenesis and *Agrobacterium*-mediated transformation of *Gladiolus hybridus* cv. ‘Advance Red’. *Plant Cell Tissue Organ Cult.* 120 717–728. 10.1007/s11240-014-0639-5

[B43] Zhu C. Li S. Qi L. Han S. (2011). *Agrobacterium tumefaciens*-mediated transformation of *Larix leptolepis* embryogenic tissue. *J. Chin. Biotechnol.* 33 75–80.

