# Supplementary material for: Stable and Efficient Agrobacterium-Mediated Genetic Transformation of Larch Using Embryogenic Callus
Source: Front Plant Sci. 2020 Nov 25;11:584492. doi: 10.3389/fpls.2020.584492 (PMC7723890; doi:10.3389/fpls.2020.584492)
Supplement: Supplementary Figure 1 — Schematic representation of binary expression vectors used in this study. [file Data_Sheet_2.docx]

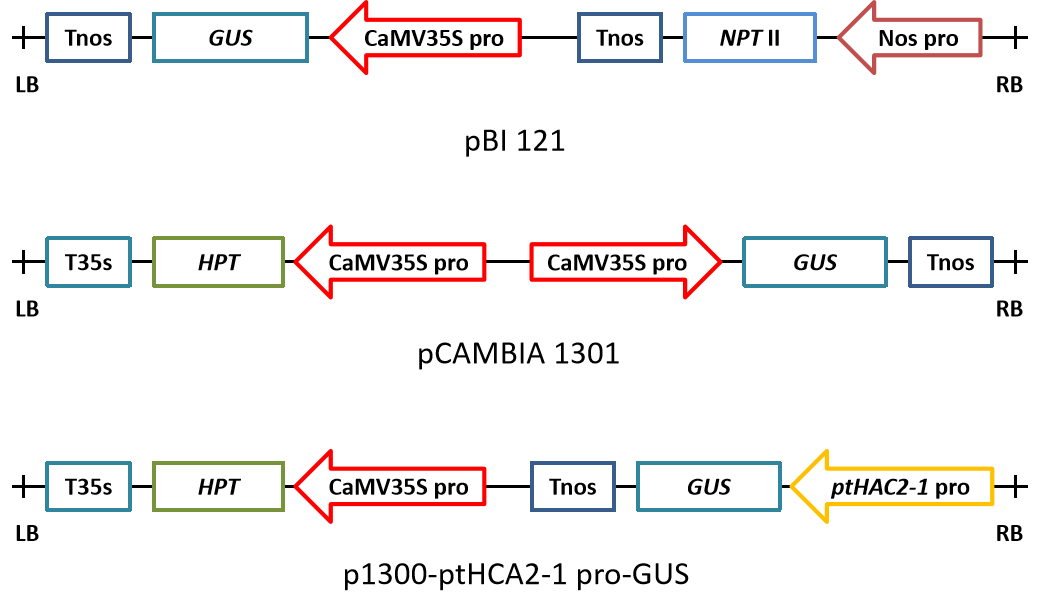


**Fig.S1 Schematic representation of binary expression vectors used in this study*.***


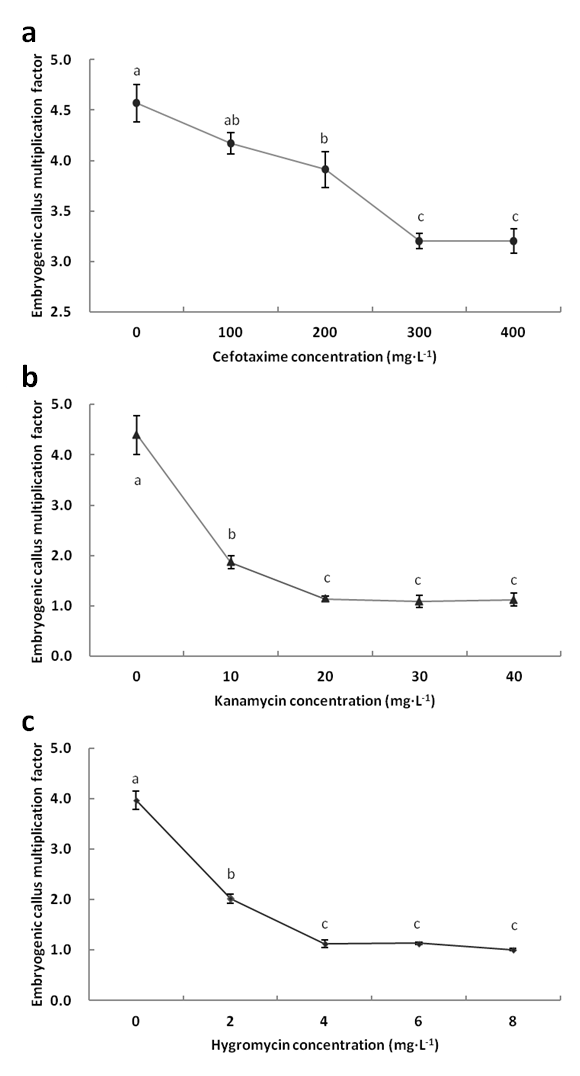


**Fig. S2. Effect of antibiotics on the proliferation of embryogenic callus of *L. olgensis*.** Effects of (**a**) cefotaxime, (**b**) kanamycin and (**c**) hygromycin concentrations on *L. olgensis* embryonic callus multiplication. The multiplication factor is the ratio of pre- and post-culture viable callus. Each value represents the mean of three independent experiments with the standard deviation (SD). Different letters indicate *p*<0.05 (Duncan’s multiple range test).


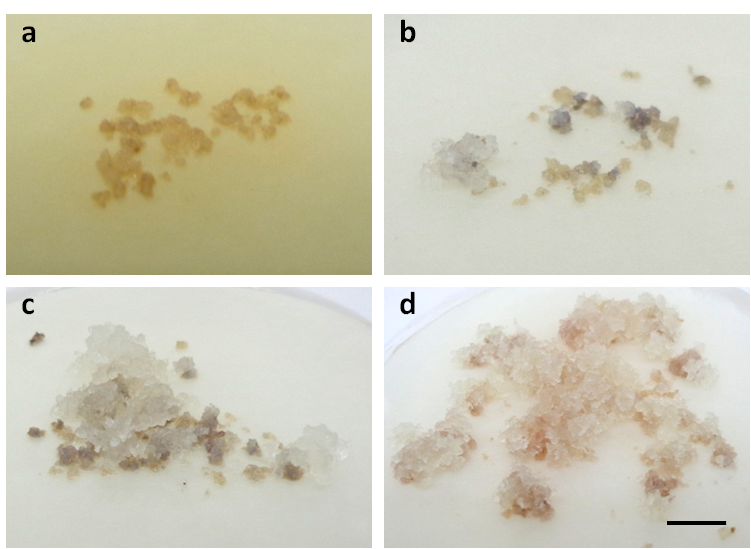


**Fig.S3 Comparison of the bacteriostatic effects of different concentrations of cefotaxime.** (**a**) The bacteriostatic effects of 0, (**b**) 100 and (**c**) 200 mg**·**L^-1^ Cefotaxime, and (d) ultrapure water (positive control) on embryonic callus of *L. olgensis* after co-culture with *A. tumefaciens* strain GV3101 (scale bar represents 1.2 cm).


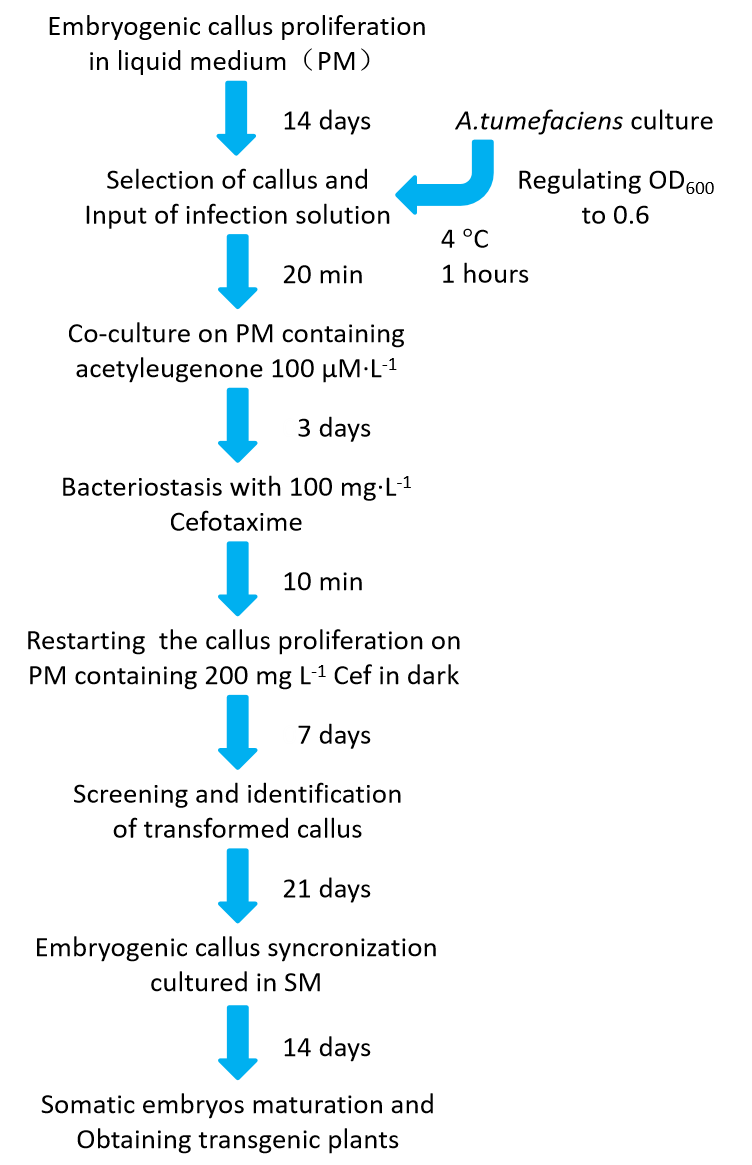


**Fig.S4. Schematic representation of the timeline for *A. tumefaciens*-mediated transformation of *L. olgensis* embryogenic callus.**


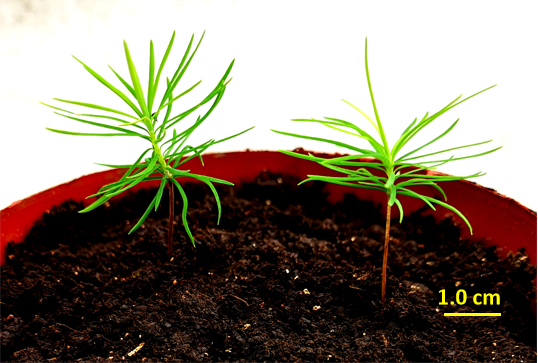


**Fig.S5. The seedling transplanting of transgenic *L. olgensis.*** After transplanting for four weeks, new needles grew on the top of the seedlings.
