# Supplementary material for: Stable and Efficient Agrobacterium-Mediated Genetic Transformation of Larch Using Embryogenic Callus
Source: Front Plant Sci. 2020 Nov 25;11:584492. doi: 10.3389/fpls.2020.584492 (PMC7723890; doi:10.3389/fpls.2020.584492)
Supplement: Supplementary Table 1 — Efficiency and conditions of Agrobacterium-mediated genetic transformation of Larix. [file Data_Sheet_1.docx]

**Table S1. Efficiency and conditions of *Agrobacterium tumefaciens****-***mediated genetic transformation of *Larix* using embryogenic callus*.***

| **Species** | **Strain** | **plasmid** | **Infection** | **Co-cultivation** | **Vir Inducers** | **Selection pressure** | **Efficiency** | **References** |
| --- | --- | --- | --- | --- | --- | --- | --- | --- |
| *Larix decidua* | GV3101 | pBI121 | OD_600_ 0.4-0.7 / 20 min | 20 ℃ / 48 h | AS (200 μM) | Geneticin (25 mg·L^-1^) | NA | Ismail *et al*., 2004 |
| *Larix leptolepis* | GV3101 | pSuper1300+ | OD_600_ 0.4 / 10 min | 20 ℃ / 48 h | AS (20 μM)0 | Hygromycin (3 mg·L^-1^) | 0.94/g | Zhu *et al*., 2011 |
| *L.kaempferi ×decidua* | C58 | pMP90 | OD_600_ 0.3 / 20 μL* | 25 ℃ / 48 h | CA (100 μM) | Kanamycin (50 mg·L^-1^) | 5/60 | Levee *et al*., 1997 |
| *L.kaempferi ×principis* | EHA1015 | pBI121 | OD_600_ 0.4 / 20 min | 19-22 ℃ / 72 h | AS (50 μM) & CA (50 μM) | Kanamycin (20 mg·L^-1^) | 8/52 | Wang, 2007 |

AS - acetosyringone, CA - coniferyl alcohol. The efficiency was calculated as (the number of resistant calli) / (total number or fresh weight of embryogenic mass) under optimum conditions. NA - specific genetic transformation efficiency is not mentioned in reference.

* twenty microlitres of the bacterial suspension were directly applied onto the embryonal masses kept on solid proliferation medium, followed by co-cultivation.

**Table S2. Composition of BM basic medium**

| **Reagent component** | | **Content /(mg·L^-1^)** |
| --- | --- | --- |
| **Inorganic ion** | NH_4_NO_3_ | 150.00 |
|  | KNO_3_ | 909.90 |
|  | KH_2_PO_4_ | 136.10 |
|  | MgSO_4_·7H_2_O | 246.50 |
|  | Mg(NO_3_)_2_·6H_2_O | 256.50 |
|  | MgCl_2_·6H_2_O | 50.00 |
|  | ZnSO_4_·7H_2_O | 14.40 |
|  | H_3_BO_3_ | 15.50 |
|  | CuSO_4_·5H_2_O | 0.125 |
|  | MnSO_4_·2H_2_O | 10.50 |
|  | CoCl_2_·6H_2_O | 0.125 |
|  | KI | 4.150 |
|  | Na_2_Mo_4_·2H_2_O | 0.125 |
|  | FeSO_4_·7H_2_O | 13.90 |
|  | Na_2_EDTA | 18.65 |
|  | CaCl_2_·4H_2_O | 50.00 |
|  | Ca(NO_3_)_2_·4H_2_O | 236.20 |
| **Organic adenda** | Inositol | 100.00 |
|  | Glycine | 002.00 |
|  | Vitamin B_1_ (Thiamine) | 001.00 |
|  | Vitamin B_5_ (Pantothenic acid) | 000.50 |
|  | Vitamin B_6_ (Pyridoxine) | 000.50 |

The basic medium (BM) used in this study was a modified BM medium (Wang *et al.*, 2009). The mother solution of macro-elements salts (×10), micro-elements salts (×100, free of Fe), iron elements (×100, ferrous), calcium elements (×100), glycine and each vitamins (×100) were prepared separately and stored in brown bottle at 4 degrees Celsius, and mixed before high temperature sterilization. Inositol was dissolved where necessary and filter sterilized.

In addition, 25 g·L^-1^ sucrose was usually added to the medium as carbon source, and 6 g·L^-1^ agar (Gel strength:1090 g/cm^2^) was used as coagulant.

**Table S3. List of primers used for PCR.**

| Gene name | Primer sequence (5’-3’) |
| --- | --- |
| GUS | GGGCGAACAGTTCCTGATTAACC |
|  | CAGTACCTTCTCTGCCGTTTCCA |
| *Npt* II | GATGTTGCTGTCTCCCAGGTCG |
|  | GCGGAGTGCATCAGGCTCTTTTC |

**Table S4. Comparison of the** **number of mature somatic embryos** **and somatic embryo germination rate of transgenic and non-transgenic embryogenic callus of *L. olgensis***

| Callus line | plasmid | Antibiotics * | Number of mature somatic embryos (ind**·**g^-1^) ** | Somatic embryos germination rate (%) |
| --- | --- | --- | --- | --- |
| WT | - | - | 211.11±20.16^a^ | 86.67±04.44^a^ |
| WT | - | Hygromycin | 0 | 0 |
| T_1_ | pBI121 | Kanamycin | 188.15±14.79^a^ | 77.04±06.79^a^ |
| T_2_ | pCAMBIA1301 | Hygromycin | 199.29±28.66^a^ | 74.82±09.25^a^ |
| T_3_ | pCAMBIA1301 | Hygromycin | 183.70±35.58^a^ | 82.96±07.14^a^ |
| T_4_ | p1300-*Pt*HCA2-1pro-GUS | Hygromycin | 210.37±15.76^a^ | 71.85±10.06^a^ |
| T_5_ | p1300-*Pt*HCA2-1pro-GUS | Hygromycin | 191.85±39.52^a^ | 69.63±12.24^a^ |

Each value represents the mean of three independent experiments with standard deviation (SD). Approximately 15 embryogenic calluses of *L. olgensis* were examined for each individual experiment. Ultrapure water was the positive control. Values with different letters indicate *P*<0.05 (Duncan‘s multiple range test).

* the concentrations of Kanamycin and Hygromycin were used in the range of 20 mg·L^-1^ and 4 mg·L^-1^ respectively.

** The number of mature somatic embryos were counted. Embryogenic calluses were cultured on synchronization medium (SM) containing 1/4 BM base salt, 60 g·L^-1^ sucrose, 15 g·L^-1^ inositol 0.5 g·L^-1^ Gln and 0.25 g·L^-1^ CH for 15 days before transferring them to maturation medium (MM).
